# National clinical guidelines: The diagnosis and management of asthma in adults

**DOI:** 10.5339/qmj.2022.fqac.12

**Published:** 2022-04-01

**Authors:** Rasmeh Al Huneiti, Bushra Saeed, Rasha Nusr, Eman Radwan, Huda Al-Katheeri

**Affiliations:** ^1^Strategic Planning and Performance Department E-mail: ralhuneiti@moph.gov.qa

**Keywords:** asthma, diagnosis and management, NCG

## Abstract

Background: Qatar has culturally diverse health professionals; and therefore, the care provided may vary according to their background, resulting in variations in care. To bridge this gap, the Ministry of Public Health (MOPH) has established the National Clinical Guidelines (NCG) Program, which aims to reduce variation in care delivery, improve value-add from the healthcare system, adopt international best practices to local context, and enable insurers and providers to access the most currently reviewed evidence-based practice in diagnosis and management of diseases.

The NCG for “Diagnosis and Management of Asthma in Adults” was developed in collaboration between Strategic Planning and Performance Department and Subject Matter Experts (SMEs) who are practicing healthcare professionals representing different healthcare organizations in Qatar.

The NCG aims to standardize the management and treatment received by adult patients with asthma across the healthcare system and adapt the best practice recommendations in the management of asthma to the culture, customs, practice, and formulary of Qatar.

Methods: This NCG has been developed through a rigorous process that aligns with international best practices and localized to the context of Qatar, involving:

• Extensive literature search for reputed published evidence specific to NCGs.
• Critical appraisal of the literature.
• Development of a baseline draft guideline.
• Review of the baseline draft by SMEs and patients.
• Review of the guidelines by the National Clinical Guidelines and Pathways Committee (NCGPC) from stakeholder organizations across Qatar.

Results: The first edition of the NCG was published on the MOPH website on December 14, 2016; and it was updated and republished on August 22, 2019. A Patient Information Leaflet (PIL) was prepared from the NCG using simple language for use by the patients. The NCG is currently under an updation process based on new evidence since August 22, 2019. A live demo was developed on how to access the NCG and its relevant pathways from the MOPH website and navigate each section of the guidelines.

Conclusion: These NCGs will improve the quality of care for patients with asthma and advocate for the best clinical practice strategies on the management of asthma in adults.

Ethical Approval: As this abstract describes the MOPH initiative, IRB is not needed, the guideline itself was approved by the NCGPC who is solely responsible for overseeing the NCG program.

Disclosure of COI: None of the authors nor NCG developers reported any conflict of interest related to the NCG.

None of the results of this NCG has been previously published in any journal or conference.
